# Applying Different Strategies of Task Constraint Manipulation in Small-Sided and Conditioned Games: How Do They Impact Physical and Tactical Demands?

**DOI:** 10.3390/s22124435

**Published:** 2022-06-11

**Authors:** João Cláudio Machado, Alberto Góes, Rodrigo Aquino, Bruno L. S. Bedo, Ronélia Viana, Mateus Rossato, Alcides Scaglia, Sérgio J. Ibáñez

**Affiliations:** 1 Faculty of Physical Education and Physiotherapy, Federal University of Amazonas, Manaus 69067-005, Brazil; jclaudio@ufam.edu.br (J.C.M.); ronelia.viana@gmail.com (R.V.); mateusrossato@ufam.edu.br (M.R.); 2 Faculty of Physical Education, State University of Campinas, Campinas 13083-859, Brazil; algj1421@gmail.com (A.G.); alcides.scaglia@fca.unicamp.br (A.S.); 3 LabSport, Post-Graduate Program in Physical Education, Center of Physical Education and Sports, Federal University of Espírito Santo, Vitória 29075-910, Brazil; aquino.rlq@gmail.com; 4 Biomechanics and Motor Control Laboratory, School of Physical Education and Sports of Ribeirão Preto, University of São Paulo, São Paulo 04024-002, Brazil; brunosbedo@gmail.com; 5 Laboratory of Sport Pedagogy (LEPE), School of Applied Sciences (FCA), State University of Campinas, Limeira 13484-350, Brazil; 6 Optimisation of Training and Sport Performance Research Group, Faculty of Sports Sciences, University of Extremadura, 06006 Badajoz, Spain

**Keywords:** soccer, task design, rules, physical demands, tactical behavior

## Abstract

This study aimed to investigate how different strategies of task constraint manipulation impact physical and tactical demands in small-sided and conditioned games (SSCG). Ten recreational U-17 soccer players participated in this study (16.89 ± 0.11 years). We used different strategies of task manipulation to design two 4 vs. 4 SSCG: Structural SSCG and Functional SSCG. In Structural SSCG, pitch format and goal sizes were manipulated, while in Functional SSCG, players were allowed to kick the ball twice and at least 5 passes to shoot at the opponent’s goal. Players participated in four Structural and Functional SSCG, of five minutes duration with a two-minute interval in between. Players’ physical performance and tactical behavior were assessed using the WIMU PRO^TM^ inertial device. Structural SSCG stimulated players to cover more distance in sprinting (*p* = 0.003) and high-speed running (*p* < 0.001). Regarding tactical behavior, Structural SSCG stimulated players to explore game space better (*p* < 0.001). Moreover, Functional SSCG stimulated players to be closer to the ball, decreasing the effective playing space (*p* = 0.008). We conclude that these strategies of task constraint manipulation impact physical and tactical demands of the game.

## 1. Introduction

Small-sided and conditioned games (SSCG) are training tasks commonly used by coaches and trainers to provide representative practice scenarios to their players and team [1]. Therefore, several studies have highlighted the importance of SSCG to improve players’ and teams’ performance, where coaches and trainers can manipulate key task constraints to emphasize specific training contents during the training sessions [2,3,4,5,6,7]. However, for these games to be considered representative training tasks, the coaches should maintain the dynamic and functional relationships between crucial sources of information and players’ actions present in the competitive environment [8]. In addition, SSCG need to be carefully adjusted to players’ skill levels and the training content emphasized by coaches and trainers [9,10,11]. Therefore, the representative training task design needs to respect the adjustment of task difficulty, complexity, and intensity levels to the player’s intrinsic dynamics [9,11,12,13].

Previous studies reported acute effects of task manipulation (e.g., number of players, the dimension and shape of the pitch, the quantity and location of goals) on players’ and teams’ performance during SSCG [2,3,4]. In addition, the impact of rule constraints can be considered a determinant to achieving the physical and tactical stimulus [12,14]. As an example, Machado et al. [12] highlighted two different strategies of task constraint manipulation: (i) modification of structural elements of the game (e.g., number of players, pitch dimension, goal sizes, etc.), and (ii) rule manipulation. The authors [12] observed that these strategies have a different impact on the tactical behavior of teams, because they were composed of players of different ages and levels of tactical skills. The teams composed of younger players and players with low tactical skills were demonstrated to have more difficulty dealing with SSCG with manipulated rules. In this regard, coaches and trainers must carefully design SSCG using the strategy of rule manipulation.

Moreover, Machado and Scaglia [15] highlighted that when the coaches and trainers manipulate structural elements of the game, the key sources of information that regulate players’ actions emerge from the game itself, i.e., from the positioning and movement of teammates and opponents, among others. However, when the coaches manipulate the rules, besides this game information, the players need to manage information from outside the game, which originates from the practitioner’s direct intervention (e.g., players can only kick the ball twice, etc.). Therefore, when the game rules are manipulated inappropriately (e.g., without considering the players’ skills level), the task difficulty and complexity may increase [12,15].

Considering that these different strategies of task manipulation might have other impacts on players’ and teams’ performance, it becomes important to understand the effects of using these different strategies on physical performance and the way players and teams structure the game space. Therefore, this study aimed to investigate how additional task constraint manipulation strategies impact physical and tactical demands in SSCG.

## 2. Materials and Methods

### 2.1. Participants

Ten U-17 recreational soccer players (16.89 ± 0.11 years) participated in this study. The players belong to a sports participation program and train together twice a week. All the procedures in this research were in accordance with the Resolution of the National Health Council (466/2012) and the Declaration of Helsinki (2013). In addition, this study was approved by the Ethics Committee in Research with Human Beings (N. 73222617.0.0000.5404).

### 2.2. Design

We applied two SSCG specifically designed to emphasize the tactical problem of maintaining ball possession, using different strategies of task constraint manipulation: (i) modification of structural elements of the game (i.e., Structural SSCG) and (ii) modification of the game through functional elements (i.e., Functional SSCG). Both SSCG have been previously used, with an emphasis on maintaining and circulating ball possession [12].

**Figure 1:**
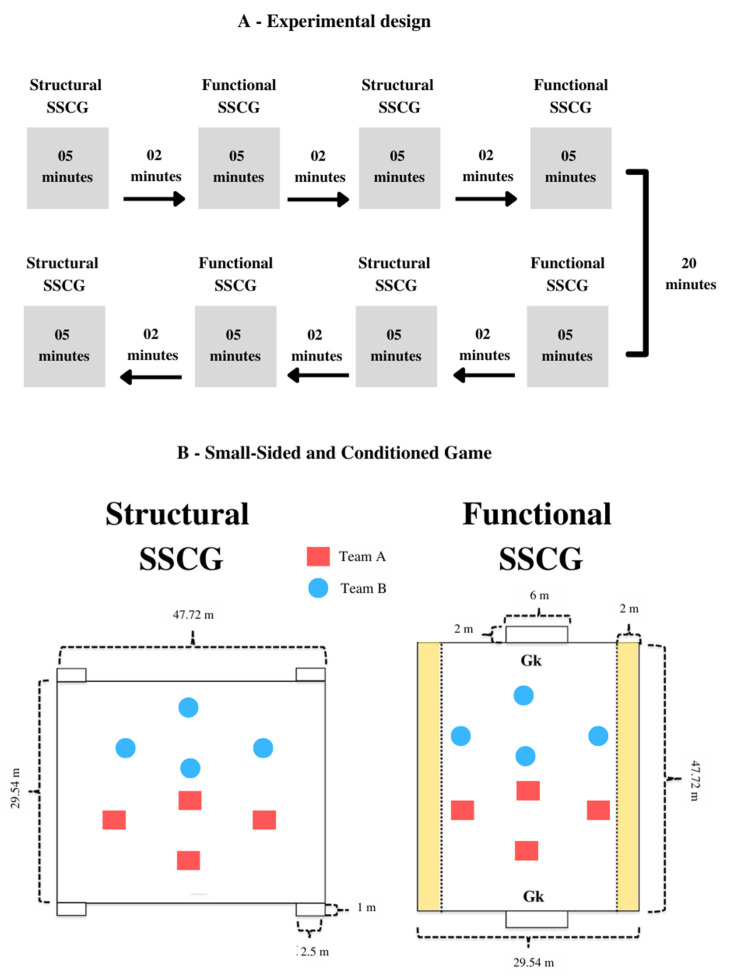
Research experimental design: (**A**) shows the order of the games applied; (**B**) shows the small-sided and conditioned games used in this study.

In the Structural SSCG, we manipulated pitch shape (wider) and goal size, and location. A 4 vs. 4 game configuration was used on a pitch measuring 47.72 m × 29.54 m (width × length), with two small goals (2.5 m × 1 m) located on both wings (Figure 1). Classical soccer rules were applied, except for offside. In the Functional SSCG, the game functional elements were modified by manipulating the rules to emphasize the tactical problem of maintaining ball possession. We used a Gk + 4 vs. 4 + Gk configuration on a pitch measuring 29.54 m × 47.72 m (width × length), with two centralized 7-a-side goals (Figure 1).

The following rules were manipulated: (i) the players were allowed to kick the ball once or twice (an extra point was awarded to the opponent’s team every time players kicked the ball more than twice); (ii) teams needed to exchange at least five passes to shoot at the opponent’s goal; (iii) an extra point was awarded to the team every time the players managed to move the ball from one wing to the other, identified from demarcated areas on the field (see Figure 1).

Goalkeepers were not allowed to participate in offensive actions, in an attempt to maintain a similar individual playing area between the two SSCG conditions (i.e., 176.2 m^2^). Four SSCG were performed in each of the conditions, with four minutes duration and two minutes interval between games. The order of the games played was randomized, as shown in Figure 1.

### 2.3. Analysis of Players’ Physical Performance

Players’ physical performance was analyzed through positional data collected using inertial devices (WIMU Pro^TM^, RealTrack System, Almería, Spain), which have been shown to be valid and reliable [16]. This device is composed of an accelerometer, gyroscope, magnetometer, and 10-Hz global position system (GPS—RealTrack System, Almería, Spain). Each participant wore a t-shirt provided by the manufacturer with a pocket to hold the GPS unit between the scapulae.

The software SPRO^TM^ (RealTrack System, Almería, Spain) was used to extract the following variables: (i) total distance covered (meters); (ii) distance covered (m) sprinting (>18 km/h^−^^1^); (iii) distance covered (m) in high-speed running (HSR—13 km/h^−^^1^ to 18 km/h^−^^1^); (iv) high acceleration (m) (>2 m/s^2^); (v) high deceleration (m) (<−2 m/s^2^); (v) the number of actions performed at a sprint. The ranges of speed were based on a previous study [17].

### 2.4. Tactical Behaviour

Studies have already used these devices to analyze tactical behaviour [18,19]. The actions performed during the games were tracked in real-time at each instant. Following the matches, data were downloaded and exported to a .*csv* file using the same version of the appropriate software (SPRO^TM^—RealTrack System, Almería, Spain) for further analysis in MATLAB scripts (The MathWorks Inc., Natick, MA, USA). Hence, the geographic coordinates were transformed to cartesian coordinates (*x*,*y*) and smoothed with a Butterworth digital filter (third-order; cut-off frequency: 0.4 Hz).

The following individual and collective tactical variables were analyzed: (i) spatial exploration index (SEI) [20]; (ii) effective playing space for each team (EPS) [20]; (iii) team width and length [20]; (iv) LpW, used to determine the length-per-width ratio per team [21]; (v) stretch index [22]. The SEI indicates players’ exploratory behavior, where higher values highlight those players that were able to explore more game space [20]. EPS considers the polygonal area of players located on the periphery of play of each team [23]. Team length represents the longitudinal distance between the most distant players, while team width represents the lateral dispersion of players [24]. The stretch index considers the average distance of each player to the team centroid, indicating how much more dispersed players are on the pitch [24]. These variables represent the individual and team space organization during the games, including the way in which players occupy game spaces through their positions and movements.

### 2.5. Statistical Procedures

Data normality distribution and homoscedasticity were verified through Shapiro–Wilk’s and Levene’s tests. To compare external load between Structural SSCG and Rules SSCG, we used a pairwise t-test. Moreover, we used both pairwise t-tests and Wilcoxon’s test to compare players’ and teams’ tactical behavior. Effect size was calculated for each pairwise comparison as follows (ES = z.√n): (i) negligible (<0.1), small (0.1–0.29), medium (0.3–0.49), and large (>0.5) [25]. We used SPSS 21.0 (Chicago, IL, USA) for statistical analysis, and the level of significance was 5% (*p* < 0.05).

## 3. Results

Regarding physical performance (Table 1), we found that players covered more distance at a sprint (*p* = 0.003) and HSR (*p* < 0.001), and also performed a greater number of sprints in Structural SSCG (*p* = 0.004). However, we did not find significant differences between game conditions (Structural and Functional SSCGs) for total distance covered (*p* = 0.301), high acceleration (*p* = 0.168), and high deceleration (*p* = 0.331).

Regarding players’ tactical behavior (Table 2), we observed that Structural SSCG stimulated players to explore more game space (SEI = *p* < 0.001). Moreover, observing EPS (*p* = 0.008) and stretch index (*p* < 0.001) variables, it was possible to note that Functional SSCG stimulated players to be closer to each other. Through team length (*p* = 0.001), width (*p* < 0.001), and LpW ratio (*p* < 0.001) measures, we observed that Structural SSCG stimulated teams to better explore the width of the pitch.

## 4. Discussion

This study aimed to investigate how different strategies of task constraint manipulation impact physical and tactical demands in SSCG. We observed that Structural SSCG stimulated players to explore game space better and stimulated teams to expand the EPS further. The rules manipulated in Functional SSCG made it difficult for the players to explore the game space, and as a result, they were able to get closer to other players. The stretch index variable also helped us to verify that players tend to get closer relative to each other in SSCG, which was designed using the strategy of rules manipulation.

Praça et al. [14] designed SSCG to emphasize progression to the target and found that players presented higher exploratory behaviors. Machado et al. [10,12] found that these manipulated rules contribute to players having more difficulty exchanging passes, keeping possession of the ball, and inhibiting players’ and teams’ exploratory behavior. The greater difficulty for players to respond to the manipulated rules resulted in players moving closer to their teammates, and behaving more statically on the field.

Regarding players’ physical performance, we found that Structural SSCG provoked more sprints than Functional SSCG. Moreover, players covered greater distances in sprinting and H.S.R. in Structural SSCG. The behavior of the prementioned tactical variables might help to understand the external load presented in these games (Structural and Functional SSCG). As players move further away from each other and as the effective playing space increases, players have more space to move in high-speed running. Other studies highlighted that when playing space increases, the distances covered by the players in different speed zones also increases [26,27]. Nunes et al. [26] observed that U-15 and U-23 players performed more sprints in SSCG with a larger playing area. Moreover, when rules were manipulated to emphasize tactical content of progression to the target, players covered greater distances in different speed zones, especially in sprinting and high-speed running [14].

In Structural SSCG, the pitch was wider, and two small goals were located on both wings. Modifying these structural elements of the game (pitch shape, goal sizes, and location) stimulated teams to expand the playing space in width. This happens as players tend to manage game space to move the ball from one wing to another to create spaces and score a goal. Even with areas restricted on both sides and with the rule that sought to stimulate the ball circulation from one wing to the other, Functional SSCG did not stimulate players to expand the game space in width.

This study aimed to raise an important discussion about the design process of SSCG in soccer, highlighting the impact of different task manipulation strategies on players’ physical performance and tactical behavior. Although it presents important information about this design process, this study has limitations that can be highlighted: a small number of players participated and it did not analyze players’ technical performance, considering whether they solved the game problems. Moreover, this study does not consider players’ initial condition, regarding their tactical skills and physical fitness. However, the results of this study are important to highlight that the exaggeration of rule manipulation negatively impacts the way players structure and move through the game space. Therefore, the design process of a representative task must consider both the strategies of task manipulation and the training content that the coach intends to emphasize.

## 5. Conclusions

We conclude that the strategies of task manipulation used impact players’ physical performance and players’ and teams’ tactical behavior differently. Structural SSCG provided a greater adequate playing space, especially in width, encouraging players to explore the pitch more. Moreover, in Structural SSCG, players performed more sprints and covered greater distances in sprinting and high-speed running speed zones. However, Functional SSCG stimulated players to get closer to their teammates. Therefore, the strategy of task modification by functional element manipulation can be used to increase game complexity level, impacting the way players and teams manage the game space. This study provides important information regarding the impact of different strategies of task manipulation, highlighting the need to carefully modify the structural elements and rules of SSCG to adjust these tasks to players’ skills level, and to the training content that coaches intend to emphasize.

## Figures and Tables

**Table 1:** Players’ physical performance in different small-sided and conditioned game conditions.

Physical Performance	Structural SSCG	Functional SSCG	*p*-Value	Effect Size
Total distance covered (m)	501.94 (48.14)	493.95 (46.12)	0.301	0.389 (medium)
Distance covered (m) at a sprint (>18 km/h^−1^)	30.52 (17.56)	10.36 (7.69)	**0.003**	1.554 (large)
Distance covered (m) in high-speed running (HSR—13 km/h^−1^ to 18 km/h^−1^)	121.82 (42.81)	77.82 (36.78)	**<0.001**	2.602 (large)
High accelerations (m) (>2 m/s^2^)	75.76 (28.67)	69.09 (19.74)	0.168	1.476 (large)
High decelerations (m) (<−2 m/s^2^)	61.31 (25.65)	56.63 (15.83)	0.331	1.141 (large)
Number of sprints	2.19 (1.22)	0.87 (0.61)	**0.004**	1.464 (large)

**Table 2:** Players’ and teams’ tactical behaviors in different small-sided and conditioned game conditions.

Tactical Behavior	Structural SSCG	Functional SSCG	*p*-Value	Effect Size
Spatial exploration index (SEI)	8.55 (1.45)	7.72 (1.43)	**<0.001**	0.584 (large)
Effective playing space (EPS)	85.97 (35.94)	67.06 (25.21)	**0.002**	0.46 (medium)
Team width	21.64 (5.09)	13.56 (3.10)	**<0.001**	1.243 (large)
Team length	16.48 (4.31)	13.56 (3.18)	**<0.001**	0.184 (small)
LpW ratio	0.78 (0.15)	0.93 (0.13)	**<0.001**	0.62 (large)
Stretch index	8.50 (2.01)	6.52 (1.25)	**<0.001**	0.761 (large)

## Data Availability

Not applicable.
